# Single-Cell RNA Sequencing Revealing Dysregulated Perturbations of Tregs in Psoriasis and Construction of a Treg-Related Diagnostic Model *via* a 101- Combination Machine Learning Computational Framework

**DOI:** 10.2174/0118715303430490251129080240

**Published:** 2026-01-12

**Authors:** Zhihao Huang, Yuan Sui, Shengxiu Liu

**Affiliations:** 1 Department of Dermatovenerology, The First Affiliated Hospital of Anhui Medical University, Hefei, Anhui, China

**Keywords:** Psoriasis, Tregs, machine learning, diagnostic model, metabolic reprogramming, single-cell RNA sequencing

## Abstract

**Introduction:**

Regulatory T cells (Tregs) exhibit compromised immunosuppressive functions in psoriasis, yet understanding of their dysregulated perturbations remains limited.

**Methods:**

scRNA-seq data from the skin of patients with psoriasis and healthy controls were analyzed to identify emigrating cells and their populations and functional states. Pseudotime as well as cell-cell communication analyses were employed to explore the origins and interactions of psoriasis-related Tregs. hdWGCNA, LASSO, and XGBoost were applied to identify critical Treg-associated gene signatures (TRGS). Various machine learning algorithms were used to develop a diagnostic model for psoriasis.

**Results:**

Psoriatic lesions displayed a significant upregulation of C4-clustered genes, such as KRT14, DDIT4, and KRT1, in the granular and spinous layers of keratinocytes, which are associated with metabolic reprogramming under localized hypoxia. Psoriasis-associated Tregs exhibited increased glycolytic activity, impairing their functionality and highlighting the IL-17-HIF-1α axis. Pseudotime analysis revealed that Treg differentiation stalls at an intermediate stage, characterized by elevated expression of LTB, IL7R, and CCL5. Tregs were found to engage in IL16-CD4 autocrine signaling, potentially enhancing their proliferation and aggregation in psoriatic lesions. Five key Treg-related genes (TRGs)—CRIP1, FBXW11, CD47, ECH1, and H3F3A—were identified, and their expression was validated in a psoriasis-mimetic cellular model. The TRGS score exhibited a significant positive correlation with patients’ PASI score (r = 0.43, *p* < 0.001). Finally, a diagnostic model was constructed based on their differential expression patterns.

**Discussion:**

This study integrated single-cell transcriptomics and machine learning approaches to reveal the heterogeneity of Tregs and their key gene signatures in psoriasis, validated the expression of these genes through *in vitro* experiments, and ultimately constructed a high-accuracy diagnostic model based on TRGs.

**Conclusion:**

This study provides a comprehensive analysis of the cellular heterogeneity of the epidermal immune microenvironment in psoriasis through single-cell transcriptomics, offering valuable insights into metabolic reprogramming, developmental pathways, cell-cell interactions, and the functional properties of Tregs in psoriasis. Additionally, a high-accuracy diagnostic model for psoriasis was developed using machine learning techniques. These findings offer a single-cell molecular perspective on immune microenvironment regulation in psoriasis and contribute to the identification of diagnostic biomarkers as well as the refinement of clinical diagnostic strategies.

## 1 INTRODUCTION

Psoriasis, a chronic inflammatory skin disorder, profoundly impacts the quality of life of affected individuals [1, 2]. It is characterized by the accelerated turnover of skin cells, resulting in the formation of thick, scaly plaques [3]. Despite considerable research into its pathophysiology [4], the precise mechanisms driving psoriasis progression remain incompletely understood. Identifying key biomarkers and elucidating disease mechanisms are essential for advancing diagnostic and therapeutic strategies [5], particularly in understanding cellular heterogeneity and targeting specific immune cell populations.

Regulatory T cells (Tregs), marked by CD4+CD25+Foxp3+ expression, are integral in maintaining immune homeostasis in psoriasis through mechanisms such as cell-contact inhibition, anti-inflammatory cytokine secretion, and metabolic regulation [6, 7]. Previous research has demonstrated significant upregulation of hypoxia-inducible factor 1α (HIF-1α) and its downstream targets in the epidermal and dermal layers of psoriatic lesions [8]. Furthermore, dysregulated activation of the IL-17A-HIF-1α axis promotes glycolysis in epidermal cells, intensifying skin inflammation and amplifying type 17 immune responses [8]. These findings suggest that the impaired immunosuppressive function of Tregs in psoriasis is likely linked to metabolic reprogramming and transcriptional heterogeneity within specific subpopulations, driven by the hypoxic microenvironment. Notably, current clinical management of psoriasis increasingly relies on biomarkers such as IL-17 and TNF-α for patient stratification and targeting biologic therapies, yet a substantial proportion of patients exhibit inadequate response [9, 10]. This highlights the need to identify more precise biomarkers and elucidate underlying functional alterations in immune subsets such as Tregs [11].

Although research has focused on Treg populations, a comprehensive characterization of their metabolic states, developmental trajectories, cell-cell interactions, and functional changes in psoriatic lesions remains lacking [12]. The integration of high-dimensional molecular data with clinical outcomes represents a promising avenue for identifying novel therapeutic targets and refining diagnostic tools [13]. Single- cell omics technologies have advanced the study of disease mechanisms and the development of precision medicine by deciphering cellular heterogeneity in diseases such as cancer and autoimmune diseases [14], and accurately identifying specific cell subsets and molecular markers [15]. This analytical capability at the single-cell resolution also serves as a key tool for exploring the phenotypic characteristics, functional dynamics, and microenvironmental interactions of regulatory T cell (Treg) subsets [16], deepening the understanding of their diversity in immune regulation. Furthermore, while machine learning (ML) algorithms such as LASSO and XGBoost have been utilized to develop predictive models [17, 18], integrating multiple algorithms allows for a more robust approach to capturing the complexity of the data, thereby enhancing biomarker identification and disease outcome prediction.

Single-cell omics technologies have advanced the study of disease mechanisms and the development of precision medicine by deciphering cellular heterogeneity in diseases such as cancer and autoimmune diseases, and accurately identifying specific cell subsets and molecular markers. This analytical capability at the single-cell resolution also serves as a key tool for exploring the phenotypic characteristics, functional dynamics, and microenvironmental interactions of regulatory T cell (Treg) subsets, deepening the understanding of their diversity in immune regulation.

This study presents a comprehensive analysis of single- cell transcriptomic data derived from skin tissues of patients with psoriasis. It identified psoriasis-related Treg subpopulations, characterizing their molecular profiles and biological functions, including metabolic alterations and developmental states. By combining hdWGCNA with ML techniques, five hub Treg-related genes (TRGs: CRIP1, FBXW11, CD47, ECH1, and H3F3A) with diagnostic significance for patients with psoriasis were identified. Our findings highlight the critical role of these genes in psoriasis progression and provide valuable insights into the development of personalized diagnostic models to enhance patient care.

## 2 MATERIALS AND METHODS

### 2.1 Data Acquisition and Processing

Four publicly available datasets were retrieved from the NCBI GEO database (http://www.ncbi.nlm.nih.gov/geo/), including the scRNA-Seq dataset GSE151177, which was selected to examine T cell population heterogeneity in human psoriasis skin tissues [19]. For scRNA-Seq data processing, the Seurat R package (version 4.3.0) was employed, following the procedures outlined in previous studies [20]. Briefly, cells with fewer than 300 detected genes, more than 6500 genes, or mitochondrial gene expression exceeding 10% were excluded to retain high-quality cellular profiles. Raw count data were normalized and scaled using the SCTransform function, followed by Principal Component Analysis (PCA). Distinct cell clusters were identified through unsupervised clustering analysis and Uniform Manifold Approximation and Projection (UMAP). Cellular clusters were annotated based on validated marker genes or dataset-specific annotations from the source data. The scMetabolism package was used to assess the metabolic profiles of individual cell subpopulations [21]. The diagnostic model was developed and validated using three bulk RNA-seq datasets (GSE30999, GSE13355, and GSE14905), with relevant sample information presented in Table **1**.

### 2.2 Trajectory and Cell-Cell Communication Analysis

Pseudotime trajectories of T regulatory cells were identified using the DDR-Tree algorithm from the Monocle package (version 2.26.0). To explore potential interactions between T regulatory cells associated with psoriasis and other cell types, the CellChat package (version 1.6.1) was employed, using default settings and the recommended pipeline [22].

### 2.3 Enrichment Analysis

The clusterProfiler package (version 4.7.1003) was used to conduct gene set enrichment analysis (GSEA), and the functional enrichment outcomes were displayed using the GseaVis package (version 0.0.8) for differentially expressed genes (DEGs) in each cell subcluster.

### 2.4 High-Dimensional Weighted Gene Co-Expression Network Analysis (hdWGCNA)

Psoriasis-associated genes related to T regulatory cells were identified using the hdWGCNA package (version 0.1.1.9010), following the standard protocol described at https://smorabit.github.io/hdWGCNA/articles/basic_tutorial.html [23]. For each sample and cell cluster, metacells were generated by combining 50 cells into each metacell using the MetacellsByGroups function. The Seurat object was split by population of interest, and the standard hdWGCNA pipeline was applied sequentially, using default parameters for the functions TestSoftPowers, ConstructNetwork, ModuleEigengenes, ModuleConnectivity, and RunModuleUMAP.

### 2.5 Shapley Additive Explanations (SHAP) Analysis

SHAP values were utilized to identify key features influencing psoriasis onset and progression. The lightgbm and xgboost packages (versions 4.5.0 and 1.7.8.1) were used for this analysis. We established the Protein–Protein Interaction (PPI) network using the STRING database (v10.5) and visualized it with Cytoscape (version 3.8.0).

### 2.6 Cell Culture

The human immortalized epidermal keratinocyte cell line HaCaT was purchased from the American Type Culture Collection (ATCC). Cells were cultured in high-glucose Dulbecco's Modified Eagle's Medium (DMEM, Gibco) supplemented with 10% fetal bovine serum (FBS, Gibco) and 1% penicillin-streptomycin (100 U/mL penicillin, 100 μg/mL streptomycin), and maintained at 37˚C in a 5% CO_2_incubator with saturated humidity. When cells reached the logarithmic growth phase, they were passaged for subsequent experiments [24].

### 2.7 Psoriasis model Establishment

To simulate the psoriasis-related inflammatory microenvironment, HaCaT cells were treated with a mixture of five inflammatory factors (M5: IL-1α, IL-17A, IL-22, Oncostatin M [OSM], TNF-1α, all from PeproTech, USA), each at a working concentration of 10 ng/mL. The M5 mixture was added to the culture medium, and cells were continuously treated for 24 hours to establish the inflammatory model [25]. Control cells were treated with an equal volume of sterile phosphate-buffered saline (PBS).

### 2.8 Quantitative Real-Time Polymerase Chain Reaction (qPCR)

Total cellular RNA was extracted using the Trizol method (TaKaRa, Japan). Genomic DNA (gDNA) was removed, and complementary DNA (cDNA) was synthesized via reverse transcription using the Evo M-MLV RT Kit with gDNA Clean for qPCR (AG, China). qPCR was performed using the SYBR Green Premix Pro Taq HS qPCR Kit (AG, China) to detect target gene expression. Primer sequences are provided in Table **2** .

### 2.9 LOOCV-Based Diagnostic Model Development *via* Machine Learning Algorithms

A consensus model was developed by integrating ten ML algorithms: elastic net (Enet), stepwise Cox regression, random survival forest (RSF), CoxBoost, lasso regression, ridge regression, partial least squares regression for Cox (plsRcox), supervised principal components (SuperPC), generalized boosted regression modeling (GBM), and survival support vector machine (survival-SVM). The diagnostic model was constructed using 101 distinct algorithm combinations within the Leave-One-Out Cross-Validation (LOOCV) framework. The GSE13355 and GSE14905 datasets were used for training and validation, respectively.

### 2.10 Statistical Analysis

Analyses and visualizations were performed using R (v4.2.1). Pearson’s correlation coefficients were used to assess relationships between continuous variables. For quantitative data, subgroup comparisons were conducted using a two-tailed, unpaired t-test or ANOVA with Tukey’s post hoc test. All experiments were independently repeated at least three times, and data are presented as mean ± standard deviation (Mean ± SD). Statistical analysis was performed using GraphPad Prism 9 software. For two-group comparisons, normality was first assessed: normally distributed data were analyzed using an unpaired Student’s t-test, while non-normally distributed data were analyzed using the Mann-Whitney U test. A *P* value < 0.05 was considered statistically significant.

## 3 RESULTS

### 3.1 Single-Cell Transcriptional Landscape of Human Psoriatic Epidermal

**Fig. (1):**
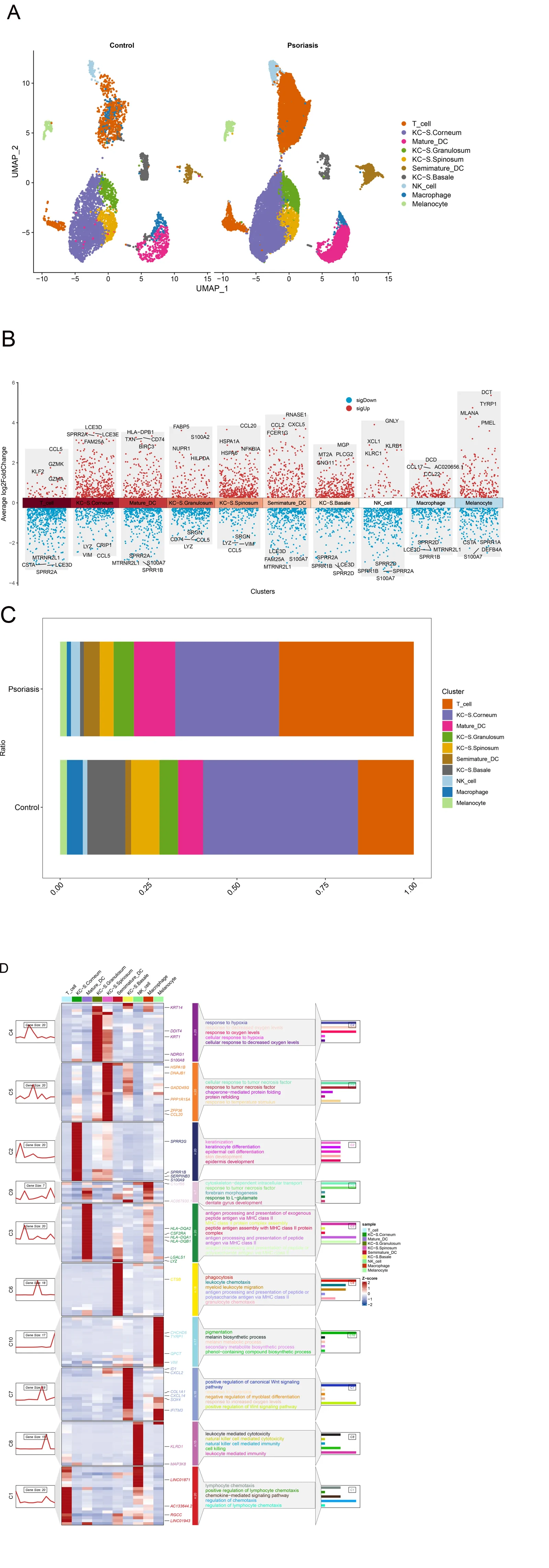
Single-cell transcriptomic profiling of human normal and psoriatic skin. (**A**) UMAP plot showing major cell types identified in normal and psoriatic skin samples. (**B**) Top 8 differentially expressed genes per cell type in psoriatic versus normal skin. (**C**) Proportional abundance of each cell population in normal and psoriatic skin. (**D**) Left: Dynamics of selected differentially expressed genes across clusters. Middle: Heatmap of representative marker genes for each cell type. Right: Heatmap of representative marker genes for each cell type.

To explore cellular heterogeneity in human psoriasis, we analyzed scRNA-seq data from psoriasis patients and healthy controls. Through cell annotation, ten major cell populations were identified: T cells, KC-S.Corneum (stratum corneum keratinocytes), Mature_DC (mature dendritic cells), KC-S.Granulosum (granular layer keratinocytes), KC-S.Spinosum (spinous layer keratinocytes), Semimature_DC (semi-mature dendritic cells), KC-S.Basale (basal layer keratinocytes), NK cells (natural killer cells), Macrophages, and Melanocytes (Figs. **1A** and **B**). Variations in cellular composition across different samples were further examined (Fig. **1C**). The analysis revealed a significant increase in the proportions of T cells and Mature_DC in psoriatic tissue compared to normal tissue, suggesting their critical roles in psoriasis progression. Additionally, key characteristic genes of each cell type were analyzed, and unsupervised clustering of these genes identified ten distinct expression trends linked to various biological functions (Fig. **1D**). Notably, genes in cluster C4, mainly composed of granular and spinous keratinocytes, showed upregulation of critical genes such as KRT14, DDIT4, and KRT1. Functional enrichment analysis indicated a significant association of these genes with hypoxia-response pathways, including “response to hypoxia” and “response to oxygen levels”. In contrast, T cell-specific genes were predominantly enriched in chemokine- mediated signaling and chemotaxis regulation pathways (Fig. **1D**).

### 3.2 Single-Cell Transcriptional Analysis Uncovered T Cell Heterogeneity in Human Psoriatic Epidermal

**Fig. (2):**
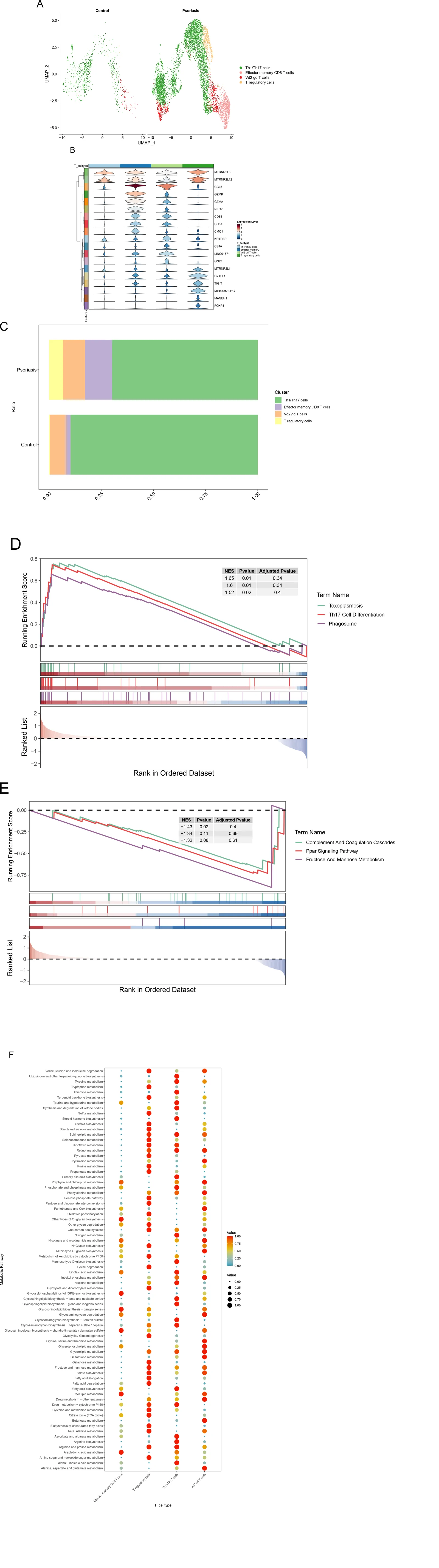
Heterogeneity of T cells in the epidermal immune microenvironment of psoriasis. (**A**) UMAP visualization of four T cell subtypes in normal and psoriatic skin. (**B**) Violin plots showing expression levels of representative differentially expressed genes across T cell subtypes. (**C**) Composition of T cell subtypes in normal and psoriatic skin. (**D** and **E**) Enriched GSEA pathways in regulatory T cells. (**F**) Heatmap of metabolic pathway activity across T cell subtypes.

Focusing on T cells, the most predominant population in the psoriatic epidermis and significantly increased compared to normal controls, this study isolated this cell population and subdivided it into four subclusters based on highly variable T cell-specific genes (Figs. **2A** and **B**). A notable enrichment of Tregs in the psoriasis group compared to normal controls was observed (Fig. **2C**), suggesting their potential pivotal role in psoriasis pathogenesis [26]. This led us to focus on Tregs in subsequent analyses.

DEGs in psoriasis-related Tregs were analyzed, followed by GSEA. For psoriasis-specific Tregs (Fig. **2D**), GSEA revealed strong enrichment in the Th17 cell differentiation pathway (NES = 1.6, *P* = 0.01) and the Toxoplasmosis pathway (NES = 1.65, *P* = 0.01), with moderate enrichment in the Phagosome pathway (NES = 1.52, *P* = 0.02). Conversely, negative enrichment was observed in the Complement and Coagulation Cascades pathway (NES = -1.43, *P* = 0.02). GSEA also suggested a potential negative correlation between Tregs and pathways involving PPAR signaling, as well as fructose and mannose metabolism (Fig. **2E**).

Given the established link between metabolic abnormalities and psoriasis progression, alterations in metabolic processes were analyzed across different T cell subtypes. The results revealed increased glycolytic activity in Tregs, which may partially explain their functional impairment. Moreover, compared to other T cell subpopulations, Tregs exhibited significant increases in tryptophan, sulfur, and pyruvate metabolism (Fig. **2F**). These results suggest that Tregs play a pivotal role in energy metabolism regulation, potentially contributing to the onset and progression of psoriasis.

### 3.3 Pseudo-Time Trajectory and Cell-Cell Communication Analysis

**Fig. (3):**
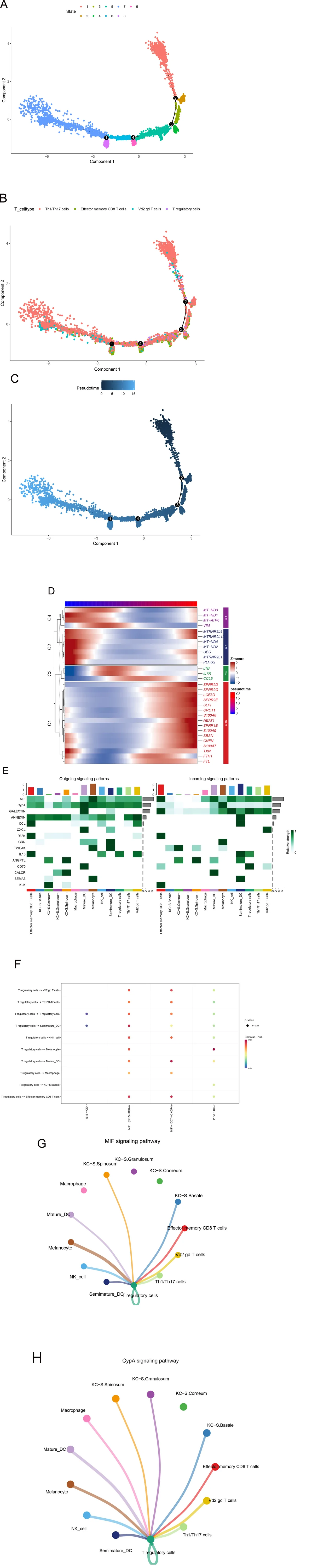
Pseudotemporal trajectory and intercellular communication analysis of Tregs. (**A-C**) Developmental trajectory of Tregs, colored by (**A**) cellular states, (**B**) associated subpopulations, and (**C**) pseudotime progression. (**D**) Heatmap of gene expression dynamics along pseudotime. (**E**) Heatmap of gene expression dynamics along pseudotime. (**F**) Bubble plot of ligand-receptor interactions between Tregs and other immune cell populations. (**G**) Circle plot of MIF signaling network interactions. (**H**) Circle plot of CypA signaling network interactions.

To investigate the origins of psoriasis-associated Tregs during disease progression, pseudotime trajectory analysis was performed on T cell subpopulations. The analysis revealed a distinct positioning of Tregs along the developmental trajectory (Figs. **3A**-**C**). Notably, Tregs were most prominently found at the intermediate stages of the trajectory, particularly at branches 2, 3, and 4. This suggests that, in psoriasis, certain signaling pathways or microenvironmental factors may disrupt Tregs, causing them to become arrested at an intermediate stage. As a result, these cells fail to attain their mature and stable immunoregulatory functions, thereby contributing to disease progression (Figs. **3A**-**C**). Further examination of gene expression patterns along the trajectory revealed that key Treg-associated genes, such as LTB, IL7R, and CCL_5_, were predominantly expressed during the intermediate stages (Fig. **3D**).

Cell communication network analysis identified robust interactions among 14 cell types, with the MIF ligand-receptor signaling pathway being the most frequent (Figs. **3E**-**H**). All cell types, except KC-S.Basale and Macrophages, acted as MIF signal senders. KC-S.Granulosum exhibited minimal MIF-sending capacity, and its signals were not received by Tregs (Fig. **3G**). The CypA signaling pathway ranked second in frequency, with all 14 cell types acting as CypA senders. Tregs received CypA signals from all cell types except KC-S.Corneum (Fig. **3H**). Tregs also functioned as both senders and receivers of IL16-CD4 signals (Figs. **3E** and **F**). Notably, this autocrine IL16-CD4 signaling axis plays a critical role in Treg proliferation and aggregation within psoriatic skin lesions. Additionally, Tregs received CD70 signals, with Mature_DC identified as the exclusive sender of this pathway.

### 3.4 Multiple Machine Learning Approaches Find the Gene Signature Pertaining to Tregs

**Fig. (4):**
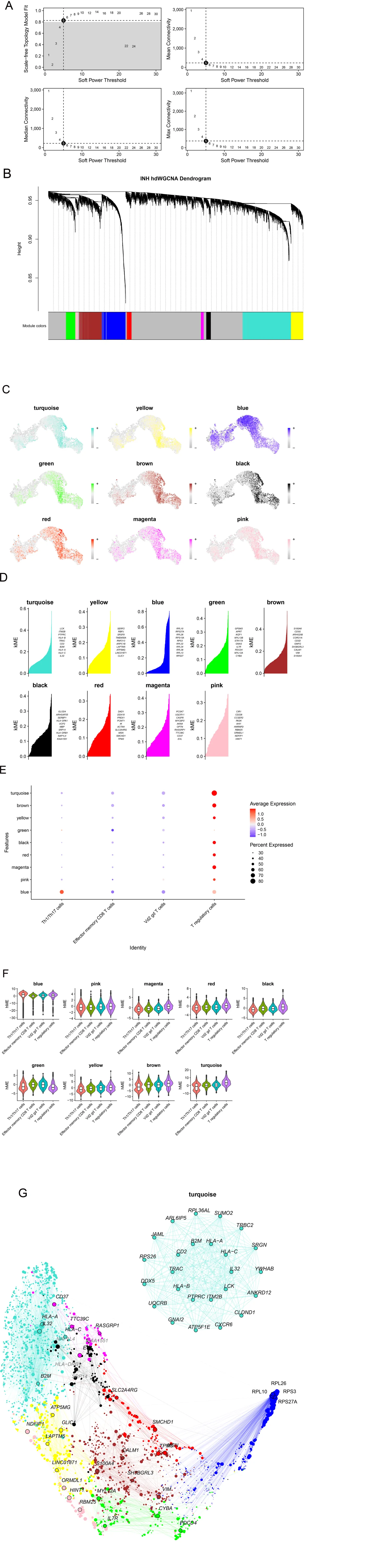
Identification of potential T regulatory cell-related module hub genes using hdWGCNA. (**A**) Soft - threshold selection for optimal network construction. (**B**) Gene module clustering diagram. (**C**) UMAP of hub gene expression across T cell clusters. (**D**) The top 10 eigengenes of each module, ranked by kME. (**E** and **F**) Module activity in distinct T cell clusters. (**G**) UMAP plot showing the co-expression network in each module.

HdWGCNA was employed to identify key modules in Tregs associated with psoriasis [27]. Optimal connectivity was achieved when the scale-free topological fit index reached 0.90, and the soft-threshold power was set to 5 (Figs. **4A** and **B**). Nine gene modules were identified, with the turquoise module showing the strongest correlation with target genes and the largest proportion of coexpressed cells (Figs. **4C**-**F**). Fig. (**4G**) displays the top four hub genes for each of the nine modules, along with their respective PPI networks.

**Fig. (5):**
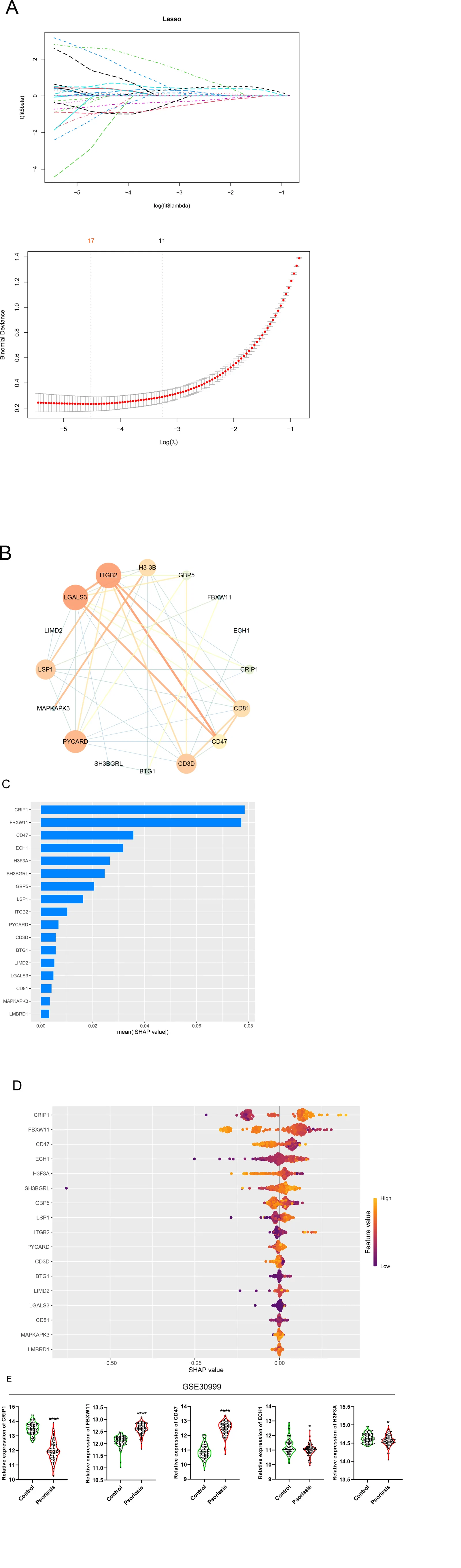
Identification of T regulatory cell-associated hub genes via machine learning. (**A**) LASSO regression analysis for feature selection of psoriasis-related Treg genes. (**B**) PPI network of 17 identified genes. (**C** and **D**) Gene feature importance analysis based on the XGBoost model, with results presented using SHAP values. (**E**) Expression levels of the top five T regulatory cell-related genes (TRGs) in psoriatic *versus* normal skin (dataset GSE30999; means ± SD, ***P < 0.001, *P < 0.05 *vs.* control).

**Fig. (6):**
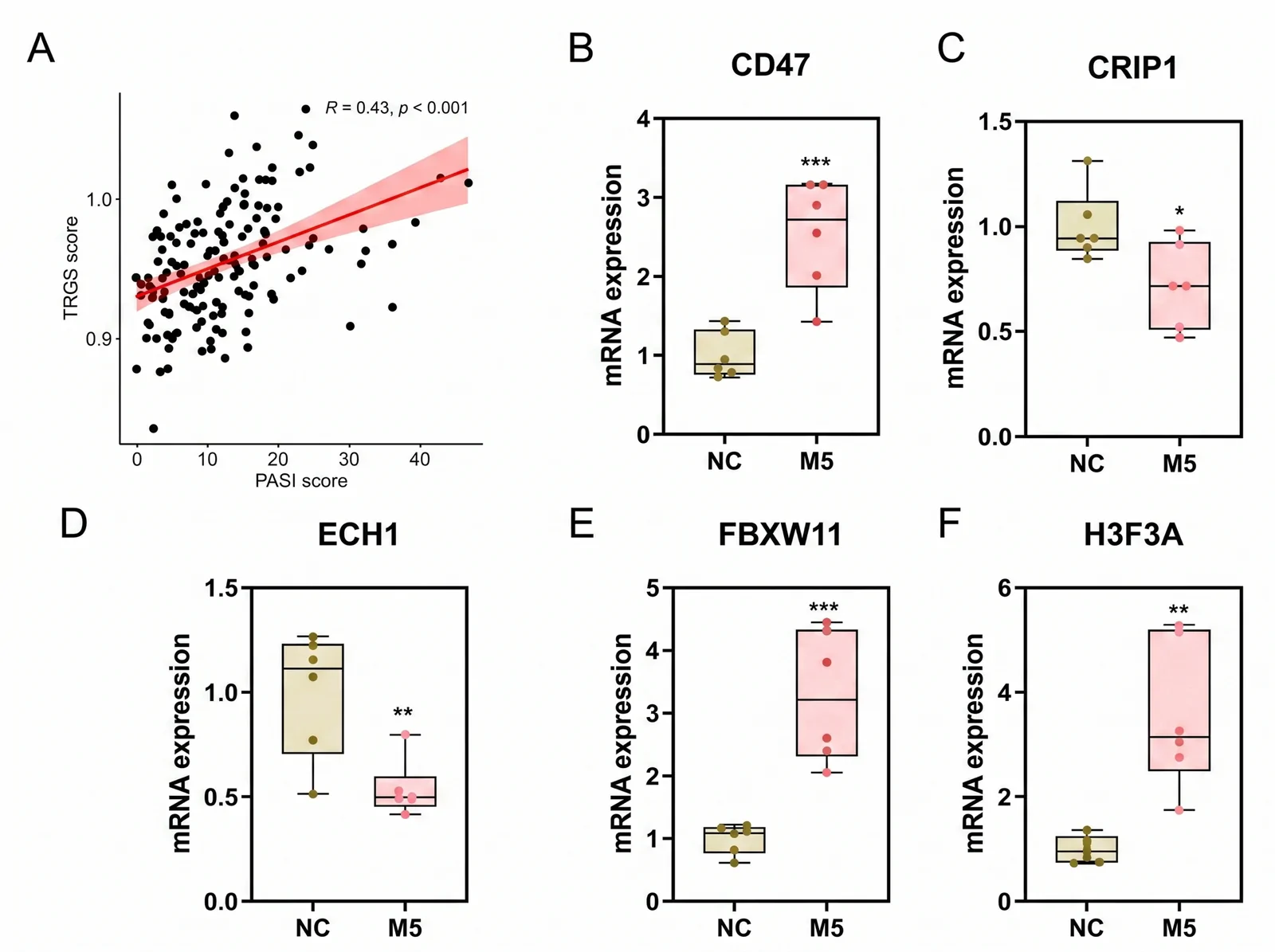
Correlation analysis between TRGs and PASI scores and validation of TRGs expression in a simulated cell model of psoriasis. (**A**) Pearson correlation analysis of PASI and TRG scores in psoriasis patients (GSE85034 dataset). (**B-F**) qPCR analysis of target gene mRNA expression in control group and M5-treated HaCaT cells, with panels B-F corresponding to genes: CD47, CRIP1, ECH1, FBXW11, H3F3A.

To further pinpoint core genes linked to psoriasis-related Tregs, the selection of relevant genes was refined using two ML algorithms: LASSO regression and the SHAP method [28, 29]. These approaches were integrated with hdWGCNA to prioritize critical features among all genes associated with Tregs. LASSO regression analysis, applied to an external dataset (GSE30999), identified 17 TRGs strongly correlated with psoriasis pathogenesis (Fig. **5A**). A PPI network was constructed to explore interactions among these genes (Fig. **5B**). Concurrently, SHAP analysis ranked all TRGs by importance, extracting the top 5 most significant genes: CRIP1, FBXW11, CD47, ECH1, and H3F3A (Figs. **5C** and **D**). Differential expression analysis revealed that FBXW11, CD47, and H3F3A were significantly upregulated in the psoriasis group compared to controls, while CRIP1 and ECH1 were downregulated (Fig. **5E**). Moreover, our findings revealed a strong positive correlation between the PASI Scores and TRGS scores in psoriasis patients (r = 0.43, *P* < 0.001) (Fig. **6A**). These findings highlight the diagnostic potential of these TRGs in psoriasis pathogenesis and their relevance to Treg functionality.

### 3.5 Validation of Expression of TRGs in a Psoriatic Inflammatory Model

To validate TRG expression patterns in a psoriasis-relevant cellular context, we performed qPCR in M5-treated HaCaT cells (M5: a psoriasis-mimetic inflammatory cytokine cocktail) compared with control cells. Results showed significant upregulation of CD47, FBXW11, and H3F3A, and downregulation of CRIP1 and ECH1 (all *P *< 0.05) (Figs. ****6B**** -**F**). These findings confirm differential TRG expression in the psoriasis-mimetic model and support their potential as critical regulators of psoriasis pathogenesis and regulatory T cell function.

### 3.6 Construction of a Psoriasis Diagnostic Model *via* Machine Learning Methods Utilizing Tregs-Related Gene Signatures

**Fig. (7):**
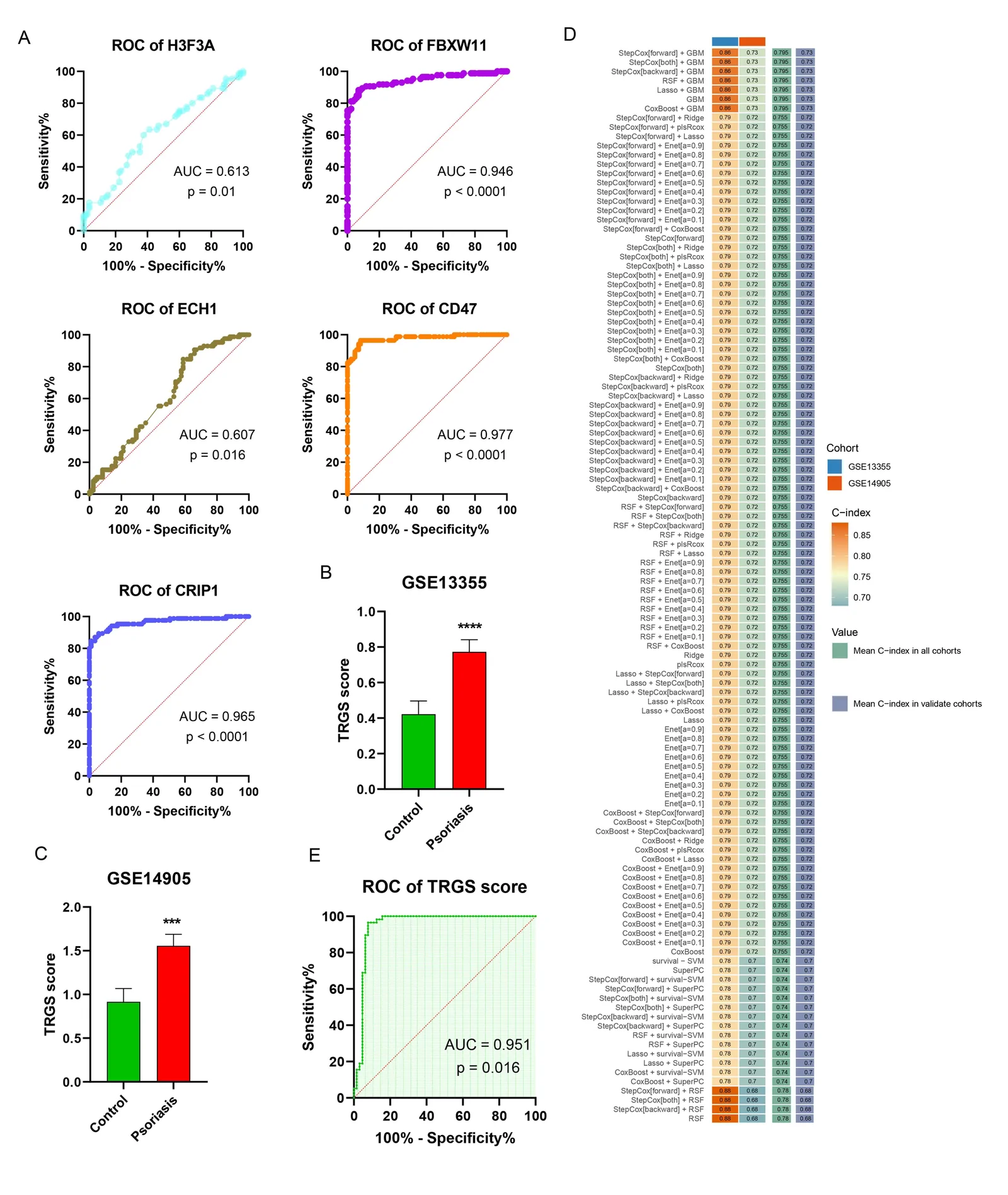
Development and validation of a consensus TRGS score by integrating bulk sequencing data with advanced machine learning techniques. (**A**) ROC curve for the identified top 5 hub T regulatory cell-related genes. (**B** and **C**) Boxplots of TRGS score in psoriasis *vs*. normal skin. (**D**) Performance of 101 prediction models evaluated by LOOCV; C-index in training and validation sets. (**E**) ROC curve of the final model in the external validation cohort (GSE30999).

Following the identification of five pivotal TRGs with significant diagnostic potential in psoriasis, ROC curve analysis was conducted to assess the diagnostic accuracy of these hub genes. The results revealed that the areas under the curve (AUCs) for FBXW11, CD47, and CRIP1 exceeded 0.9, confirming the robustness of the five-gene signature as a reliable discriminator for patients with psoriasis (Fig. **7A**). Subsequently, the TRG signature (TRGS) score was evaluated in both control and psoriasis tissues, demonstrating a significant increase in the TRGS score within psoriasis tissue (Figs. **7B** and **C**). An ML-integrated approach was then developed to compute TRGS scores using external bulk RNA-seq datasets. Through LOOCV, 101 combined ML models were evaluated, with GSE13355 serving as the training dataset and GSE14905 as the validation set. The concordance index (C-index) was calculated for each model, and the optimal model was identified as StepCox (forward) + GBM (Fig. **7D**). StepCox + GBM consistently achieved the highest C-index across both the training cohort (GSE13355, C-index =0.8645) and the external validation cohort (GSE14905, C-index = 0.7341) compared to other candidate top-performing models (Table **S1**). Validation with GSE30999 yielded an AUC of 0.951 (Fig. **7E**), further substantiating the model's strong discriminatory power between normal and psoriasis samples.

## 4 DISCUSSION

Psoriasis, a chronic inflammatory disorder, is strongly influenced by genetic factors and can be triggered by environmental conditions [30]. While Treg dysfunction in psoriasis is linked to disease progression [31], the understanding of their dysregulated perturbations remains limited. In this study, an in-depth single-cell transcriptional analysis of the human psoriatic epidermis was performed, identifying and characterizing Treg subpopulations. The cellular heterogeneity of the psoriatic epidermal immune microenvironment was explored through functional enrichment analysis, pathway and metabolic characterization, differential expression analysis, pseudotime trajectory analysis, and cellular communication analysis. Additionally, ten ML algorithms and 101 algorithmic combinations were integrated to construct and validate an optimized diagnostic model, providing new insights and potential clinical tools for psoriasis management.

Our study found a significant increase in Treg abundance in psoriasis samples, but their development was largely arrested at an intermediate stage. Compared to normal skin tissues, Tregs in the psoriatic epidermal immune microenvironment exhibited notable differences in functional activity and metabolic state. These changes were closely associated with enhanced glycolytic activity in Tregs under hypoxic conditions within the inflammatory microenvironment. Cell communication analysis further revealed that abnormal activation of multiple signaling pathways, including MIF/CD74/CXCR4, MIF/CD74/CD44, CyPA, and the self- sustaining IL-16-CD4 autocrine loop in Tregs, may collectively contribute to the pathogenesis of psoriasis, suggesting these pathways as potential therapeutic targets. Furthermore, this study identified a novel set of diagnostic biomarkers, and their expression was validated in a psoriasis-mimetic cellular model. A diagnostic model was further constructed based on their differential expression patterns. The model demonstrates strong clinical potential. Compared to traditional pathological diagnostic methods, its objective assessment based on gene expression data helps reduce errors caused by subjective physician judgment [32], while its high sensitivity makes it a promising tool for early screening and auxiliary diagnosis of psoriasis [33].

Previous studies have highlighted the significant role of T cells in psoriasis and associated mental health issues [34], but few have explored the diversity of T cell populations in psoriasis. In our analysis, the proportion of T cells in psoriatic skin tissue was significantly elevated, particularly for Tregs, which were nearly absent in the control group but abundant in psoriasis samples.

Nearly all studies, however, report increased infiltration of Tregs in psoriatic lesional skin compared to healthy donors, which aligns with the current findings [35-38]. Tregs were identified as both senders and receivers of IL-16-CD4 signaling. Upon extracellular secretion in a multimeric form, IL-16 binds to the CD4 receptor on the cell surface, activating downstream PI3K/Akt and STAT6 pathways, thereby inducing chemotactic responses [39]. Beyond attracting Th1 cells, IL-16 can also prompt Tregs to migrate to inflamed or damaged areas, enhancing local immune cell aggregation and contributing to the pro-inflammatory immune response [40]. This self-sustaining IL-16-CD4 autocrine loop plays a pivotal role in promoting the expansion of Treg cells in psoriatic lesions. Additionally, the CD70-CD27 pathway likely supports the survival and proliferation of psoriatic Tregs. Tregs receive CD70 signals, with mature DC identified as the exclusive sender. CD70 is a ligand for CD27, which regulates cell proliferation and survival by binding to TRAFs and activating signaling pathways such as NF-κB and c-Jun N-terminal kinase [41, 42]. CD27-CD70 co-stimulation supports the survival of immature Tregs in the thymus [43, 44]. A significant proportion of cutaneous Tregs originates from the thymus [45].

Tregs exhibited a negative correlation with the PPAR signaling pathway, suggesting that inhibition of this pathway may be linked to the pro-psoriasis phenotype of Tregs. Consistently, prior research has proposed that PPAR-γ agonists could serve as a therapeutic option to reduce inflammation in psoriatic skin lesions and mitigate related comorbidities [46]. Our results further revealed that Tregs displayed increased expression of genes associated with several metabolic pathways, including tryptophan, sulfur, and pyruvate metabolism. Previous studies indicate that tryptophan metabolism can modulate inflammatory gene expression and suppress inflammation [47]. While metabolic disruptions are well-documented in immune cells in psoriasis [48], the relationship between Treg-related immunology and metabolism remains poorly understood, warranting further investigation.

Tregs exhibited metabolic flexibility under varying conditions. Traditionally, Tregs were believed to primarily rely on oxidative phosphorylation (OXPHOS) and fatty acid oxidation for energy production. However, GO enrichment analysis revealed that, under hypoxic conditions, as in the psoriatic inflammatory microenvironment, Tregs showed increased glycolytic activity (Fig. **2F**). IL-17-driven epithelial HIF-1α activation positively regulates glycolysis in psoriasis [8]. HIF-1α is known to regulate the transcriptional program of glycolysis [49], and IL-17 activates HIF-1α *via* the ERK1/2-AKT-mTOR pathway, promoting glycolysis in epithelial cells [50]. This enhanced glycolysis in Tregs rapidly provides energy but also leads to functional inhibition and phenotypic instability [51]. Moreover, local hypoxia in psoriatic skin lesions, coupled with an inflammatory microenvironment enriched in factors such as IL-6 and IL-23, further inhibits Treg activity [52]. In contrast to previous studies focusing on the IL-17-HIF-1α axis in promoting glycolysis within epithelial cells in psoriasis [8], our findings suggest its potential role in similarly modulating skin Treg cells. Specifically, HIF-1α activation may enhance glycolytic activity in Tregs, and this metabolic reprogramming could impair their immunosuppressive function, ultimately contributing to disrupted local immune homeostasis and exacerbated inflammation.

Tregs play a critical role in immune system regulation through their secretory functions [53]. scRNA-seq offers significant opportunities to explore the interaction network between Tregs and other cell types in psoriatic skin tissue. Our analysis revealed that Tregs interact with other cell types through inflammation-related signaling pathways, such as the MIF/CD74/CXCR4, MIF/CD74/CD44, and CyPA pathways, which could serve as potential therapeutic targets for psoriasis.

This study employed hdWGCNA, a sophisticated bioinformatics tool for detecting cell-related gene modules, in combination with LASSO and SHAP analyses, to establish a diagnostic gene signature associated with Tregs. The identification of potential diagnostic biomarkers, including CRIP1, FBXW11, CD47, ECH1, and H3F3A, represents a significant advancement in the diagnosis and monitoring of psoriasis. While CD47, a receptor from the immunoglobulin superfamily involved in regulating cell homeostasis and immune functions [54], is well documented, the roles of other identified genes in psoriasis remain largely unexplored and warrant further investigation. Notably, the identified TRGS offers substantial clinical potential for diagnosing psoriasis.

To enhance psoriasis diagnosis, an ML model was developed using the LOOCV framework [55], ensuring consistent model performance. The StepCox (forward) + GBM ML algorithm emerged as the preferred model. Further validation with independent cohorts reinforced the model's robustness, demonstrating its potential as a reliable diagnostic tool.

While promising, our study has several limitations. First, the datasets analyzed are relatively small. Despite utilizing multiple datasets, the overall sample size may not adequately capture the heterogeneity among psoriasis patients. Larger and more diverse cohorts are needed in future studies to improve the reliability of the results, ensuring that the identified biomarkers are applicable to a broad range of populations and clinical settings. Furthermore, although the identified TRGs show promise as diagnostic biomarkers, their clinical applicability requires further validation. We plan to conduct molecular and functional experiments using psoriatic tissue samples to confirm the expression patterns of key genes and to elucidate the biological mechanisms through which they regulate Treg function.

## CONCLUSION

In conclusion, this research advances our understanding of T cell heterogeneity in human psoriasis skin. By characterizing the unique molecular and biological traits of psoriasis-associated Tregs, as well as highlighting the diagnostic value of their signature genes, these findings provide critical insights into psoriasis development. Ultimately, this knowledge could improve disease prediction and support the development of targeted treatments for patients with psoriasis.

## Figures and Tables

**Table 1:** Characteristics of analyzed datasets.

**Dataset**	**Year**	**Area**	**Species**	**Platform**	**Data Type**	**Control**	**Psoriasis**
GSE151177	2021	USA	Homo	GPL18573	scRNA-seq	5	13
GSE13355	2009	USA	Homo	GPL570	Bulk RNA-seq	122	58
GSE14905	2009	USA	Homo	GPL570	Bulk RNA-seq	49	33
GSE30999	2011	USA	Homo	GPL570	Bulk RNA-seq	85	85

**Table 2:** Primer sequences for quantitative PCR.

Genes	Forward Primer (5’-3’)	Reverse Primer (5’-3’)
CRIP1	TTTTGCCTCGGAACTGGACC	CAGGCGCGAGATGAGAGG
FBXW11	TGCTGCGCGGGGAGAG	GACCTTGGCACAGAACACATGA
CD47	GAGGAACCCCTTAATGAATAACTG	TGTTTCTTCTCCCCAACAGTGAA
ECH1	CTACTGACCCGGCGACTG	CGTCACACGAAGGGACTCAT
H3F3A	GTCTACGGCGGACTCGGG	CAGAGACCTCCTTACCCCCTT

## Data Availability

The research information can be accessed upon a valid request. Gene expression information was obtained through the GEO database, specifically utilizing datasets GSE151177, GSE30999, GSE13355, and GSE14905.

## LIST OF ABBREVIATIONS

Tregs: Regulatory T cells
TRGS: Treg-associated Gene Signatures
TRGs: Treg-related Genes
ML: Machine Learning
PCA: Principal Component Analysis
UMAP: Uniform Manifold Approximation and Projection
DEGs: Differentially Expressed Genes
GSEA: Gene Set Enrichment Analysis
hdWGCNA: High-Dimensional Weighted Gene Co-expression Network Analysis
SHAP: Shapley Additive Explanations
PPI: Protein-Protein Interaction
ATCC: American Type Culture Collection
PBS: Phosphate-Buffered Saline
LOOCV: Leave-One-Out Cross-Validation
AUCs: Areas Under the Curve
TRAFs: Tumor Necrosis Factor Receptor-Associated Factors
OXPHOS: Oxidative Phosphorylation
