# Supplementary material for: Single-Cell RNA Sequencing Revealing Dysregulated Perturbations of Tregs in Psoriasis and Construction of a Treg-Related Diagnostic Model via a 101- Combination Machine Learning Computational Framework
Source: Endocr Metab Immune Disord Drug Targets. 2026 Jan 12;26:E18715303430490. doi: 10.2174/0118715303430490251129080240 (PMC13575954; doi:10.2174/0118715303430490251129080240)
Supplement: Supplementary file 1 [file EMIDDT-26-E18715303430490_SD1.pdf]

Supplementary Material

Single-Cell RNA Sequencing Revealing Dysregulated Perturbations of Tregs in Psoriasis and Construction of a Treg-Related Diagnostic Model via a 101- Combination Machine Learning Computational Framework

Zhihao Huang[1], Yuan Sui[1] and Shengxiu Liu[1,*]

[1]*Department of Dermatovenerology, The First Affiliated Hospital of Anhui Medical University, Hefei, Anhui, China*

Table S1: Performance Metrics of Top-Performing Diagnostic Models.

**Model**	**C-index (GSE13355)**	**C-index (GSE14905)**	**95% CI (GSE13355)**	**95% CI (GSE14905)**
StepCox [forward] + GBM	0.8645	0.7341	0.802-0.927	0.628-0.840
StepCox [forward] + GBM	0.8561	0.7268	0.791-0.921	0.621-0.833
StepCox [backward] + GBM	0.8602	0.727	0.796-0.924	0.621-0.833
RSF + GBM	0.8613	0.7253	0.797-0.925	0.619-0.832
Lasso + GBM	0.8622	0.7301	0.798-0.926	0.623-0.837
GBM	0.8625	0.7281	0.799-0.926	0.622-0.834
CoxBoost + GBM	0.8607	0.7293	0.796-0.925	0.623-0.836
